# Three-Dimensional Analysis of Vocal Fold Oscillations: Correlating Superior and Medial Surface Dynamics Using Ex Vivo Human Hemilarynges

**DOI:** 10.3390/bioengineering11100977

**Published:** 2024-09-28

**Authors:** Reinhard Veltrup, Susanne Angerer, Elena Gessner, Friederike Matheis, Emily Sümmerer, Jann-Ole Henningson, Michael Döllinger, Marion Semmler

**Affiliations:** 1 University Hospital Erlangen, Medical School, Division of Phoniatrics and Pediatric Audiology, Department of Otorhinolaryngology Head and Neck Surgery, Friedrich-Alexander-Universität Erlangen-Nürnberg, 91054 Erlangen, Germany; susanne13.angerer@web.de (S.A.); elena.gessner@fau.de (E.G.); friederike.matheis@fau.de (F.M.); emily.suemmerer99@web.de (E.S.); michael.doellinger@uk-erlangen.de (M.D.); marion.semmler@uk-erlangen.de (M.S.); 2 Department of Computer Science, Friedrich-Alexander-University Erlangen-Nürnberg, 91054 Erlangen, Germany; jann-ole.henningson@fau.de

**Keywords:** hemilarynx, 3D laryngoscopy, high-speed imaging, laryngeal modeling, mucosal wave propagation, empirical eigenfunctions, vocal folds

## Abstract

The primary acoustic signal of the voice is generated by the complex oscillation of the vocal folds (VFs), whereby physicians can barely examine the medial VF surface due to its anatomical inaccessibility. In this study, we investigated possibilities to infer medial surface dynamics by analyzing correlations in the oscillatory behavior of the superior and medial VF surfaces of four human hemilarynges, each in 24 different combinations of flow rate, VF adduction, and elongation. The two surfaces were recorded synchronously during sustained phonation using two high-speed camera setups and were subsequently 3D-reconstructed. The 3D surface parameters of mean and maximum velocities and displacements and general phonation parameters were calculated. The VF oscillations were also analyzed using empirical eigenfunctions (EEFs) and mucosal wave propagation, calculated from medial surface trajectories. Strong linear correlations were found between the 3D parameters of the superior and medial VF surfaces, ranging from 0.8 to 0.95. The linear regressions showed similar values for the maximum velocities at all hemilarynges (0.69–0.9), indicating the most promising parameter for predicting the medial surface. Since excessive VF velocities are suspected to cause phono-trauma and VF polyps, this parameter could provide added value to laryngeal diagnostics in the future.

## 1. Introduction

The human voice is crucial for communication and self-expression in personal and professional life [1,2]. Vocal fold (VF) oscillation in the larynx produces the primary acoustic signal [3], vibrating between 100 Hz and 300 Hz during normal phonation [4] and can exceed 1500 Hz during singing [5], which is then modulated in the vocal tract and radiated from the mouth. The oscillation results from complex interactions between airflow from the lungs, muscle tension, and the elastic properties of the VFs. As air is emitted from the lungs, it passes through the gap between the two VFs (glottis), causing them to open and close rapidly in a wave-like motion. Normal VF oscillations are assumed to be periodic and symmetric, with complete glottal closure within each cycle being ideal, though often not entirely fulfilled [6,7].

**Figure 1:**
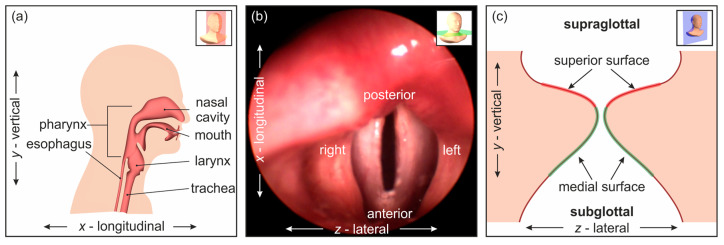
(**a**) Sagittal section of the upper respiratory tract; (**b**) view of the transverse plane through the upper respiratory tract, showing the vocal folds in the larynx; (**c**) cross-section view of the vocal folds (frontal plane). Note that the medial VF surface typically cannot be examined by laryngoscopy.

The visual assessment of the laryngeal region typically employs oral or nasal laryngoscopy (see Figure 1a), using a rigid or flexible endoscope to examine the pharynx, larynx, and VFs (see Figure 1b). High-speed video endoscopy (HSV) captures the high-frequency oscillations of the VFs with recording rates exceeding 4000 frames per second [8]. While traditional laryngoscopy provides two-dimensional (2D) images, three-dimensional (3D) data of VF oscillations are believed to enhance diagnostic accuracy [9,10,11,12]. However, current 3D laryngoscopy techniques are also limited to capturing data from the superior VF surface, leaving the medial surface unexamined (see Figure 1c).

To address this limitation, our research aims to infer the medial VF surface’s dynamic behavior from the superior surface’s oscillation patterns, focusing on human laryngeal tissue for clinically relevant insights. We previously developed a cadaver hemilarynx measurement setup, allowing comprehensive VF analysis by bisecting the larynx and providing a clear view of the complete VF surface [13].

In the present study, we expanded upon our previous work by recording the medial and superior VF surfaces of four human hemilarynges synchronously during sustained phonation using two high-speed cameras from different angles and reconstructing the 3D coordinates. Each hemilarynx was tested under 24 different conditions, varying mean flow rates, and variable laryngeal configurations, resulting in 96 analyzed VF oscillation configurations. Comparable parameters, including velocities and displacements of the VF surfaces, were derived from the 3D datasets.

This expanded dataset aims to investigate relationships in the oscillatory behavior between the superior and medial VF surfaces and highlight interindividual differences/consistency. By validating the functionality of our measurement setup with a larger sample size, this research contributes to a deeper understanding of VF dynamics and advances the potential applications for 3D laryngoscopy.

A significant contribution of this research is the open accessibility of a comprehensive 3D dataset of human VF oscillations. Voice research often suffers from a lack of experimental data to train and verify models of VF movement, with many studies relying on animal [14] or synthetic larynges [15], which may not entirely replicate human VF behavior. By making this dataset freely available, we aim to support developing and validating more accurate models in the voice research community.

## 2. Materials and Methods

### 2.1. Hemilarynges

A total of four hemilarynges was analyzed in this study. The body donors’ basic information and the respective identifiers used in this study are shown in Table 1. The data from hemilarynx HL1, presented in a previous study, were expanded with three additional datasets ranging from HL2 to HL4, which were analyzed identically.

**Figure 2:**
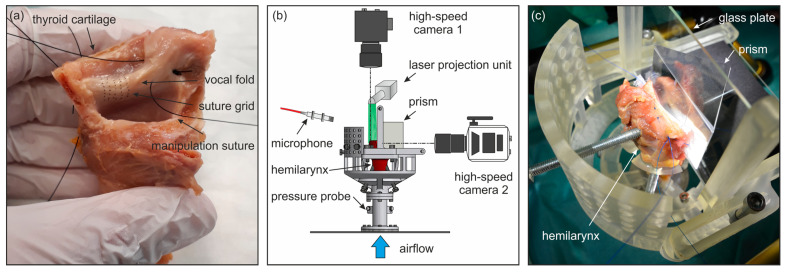
(**a**) Prepared hemilarynx with sewn-in marker points for 3D reconstruction by stereovision; (**b**) principle drawing of the measurement setup; (**c**) hemilarynx in 3D-printed larynx mount.

The preparation process was conducted identically for all hemilarynges, as described by Veltrup et al. [13]. The larynges were stored at −70 °C and then thawed overnight at 5 °C in NaCl before the experiment. During the preparation process, the left side of the thyroid cartilage (TC), arytenoid cartilage (AC), and vocal folds (VFs) were removed (see Figure 2a). On the remaining right VF, a rectangular array of 35 knots was sewn onto the medial VF surface using Ethilon 9-0 nylon sutures (Ethicon, Inc., Bridgewater, NJ, USA), which served as marker points for 3D reconstruction. One hemilarynx was analyzed per day. During the experiments, we utilized a coordinate system, where x refers to the longitudinal direction, y to the vertical direction, and z to the mediolateral direction, as indicated in Figure 1. Two types of manipulations were applied to the hemilarynx: elongation and adduction. Elongation was induced by attaching weights to a suture connected to the thyroid cartilage. Adduction was achieved by two sutures with weights passed through the arytenoid cartilage, which were manipulated in opposing directions to generate torque on the AC.

### 2.2. Measurement Setup

The measurement setup was identical to the one described by Veltrup et al. [13]. It was based on previous research by Döllinger et al. [16]. It included a mount for the hemilarynx, two high-speed camera systems, and a data acquisition system with a microphone and a subglottal pressure probe (see Figure 2b). The setup featured a modular 3D-printed hemilarynx mount made from resin (see Figure 2c), designed to fit precisely onto the artificial trachea, which initiated the VF oscillation via controlled airflow. Subglottal pressure was measured using an XCS-93 pressure probe (Kulite Semiconductor Products, Inc., Leonia, NJ, USA) flush-mounted onto the internal wall of the artificial trachea, and the radiated sound was recorded using a 4189 1/2 inch free-field microphone (Brüel & Kjær, 2850 Nærum, Denmark) placed 15 cm from the hemilarynx at an angle of approximately 30°. The hemilarynx was fixated using screws on the cricoid cartilage from below. A vertical glass plate, aligned with the glottal midline, allowed the observation of the medial VF surface (see Figure 2c). The contact surfaces between the hemilarynx and the glass plates were sealed using gauze strips and dental bonding paste. During the measurements, the VF did not make contact with the glass plate to avoid visual obstruction by contaminating the glass. The setup included two synchronized high-speed cameras for simultaneous top-down and side-perspective recordings of the VF oscillation. The superior VF surface was captured using an Os-8 high-speed camera (Integrated Design Tools, Inc., Los Angeles, CA, USA). A green (532 nm) laser grid was projected onto the VF from above for 3D reconstruction. This setup provided a resolution of 1200 × 800 px with 24 px per mm, a focal depth of 3 cm, and a laser dot diameter between 0.2 mm and 0.5 mm, ensuring an accuracy of 0.15 mm. The medial VF surface was captured from the side using a v2511 high-speed camera (Vision Research, Wayne, NJ, USA) with a prism to produce a stereoscopic image, allowing 3D reconstruction using the sewn-in marker points. This setup offered a resolution of 768 × 768 px with 15 pixels per mm at a focal depth of 1 cm and an accuracy of approximately 5%. Both cameras were synchronized at a frame rate of 4 kHz, and all measurements were triggered simultaneously, ensuring synchronous video and analog data. A mass flow controller controlled the airflow, initiating VF oscillation, and air humidity was increased with a humidifier to prevent VF tissue dehydration.

### 2.3. Measurement Protocol

The hemilarynx was examined within approximately four hours after preparation. The influence of three measurement variables was investigated: VF elongation, VF adduction, and flow rate. The three methods were selected since they are the most impactful imitable manipulations used in vivo to modify the voice. The flow rate was used to manipulate the oscillation amplitude of the VFs and, thus, the volume. Elongation and adduction were used to adjust the tension, shape, and position of the VF. In addition, they were selected because they are frequently used in ex vivo studies, which allows the results to be compared with the literature. For elongation, 10 g and 20 g weights were used on the thyroid cartilage. For adduction, 10 g, 20 g, and 50 g weights were applied to threads acting on the arytenoid cartilage. VF oscillation was initiated at four flow rates, starting with the onset flow and three incremental increases of 5 SLM steps, resulting in 24 individual measurements per hemilarynx.

### 2.4. Data Analysis

#### 2.4.1. Three-Dimensional Reconstruction

**Figure 3:**
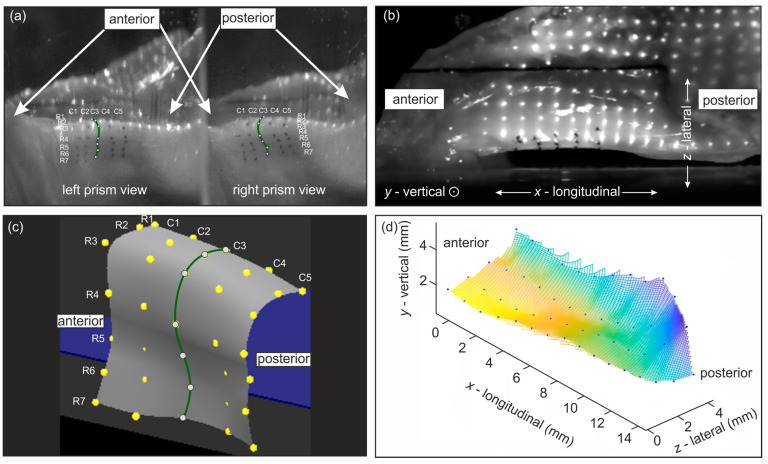
(**a**) Medial surface of prepared hemilarynx with sewn-in marker points viewed through a prism yielding two views of the surface allowing for 3D reconstruction [19]; (**b**) superior surface of hemilarynx with projected laser points; (**c**) reconstructed 3D points of sutures on the medial surface; (**d**) reconstructed 3D laser points on the superior surface.

The 3D reconstruction of the medial VF surface followed the method by Döllinger et al. [16]. Using a prism, the hemilarynx was observed from two perspectives using one camera (see Figure 3a). Calibration was performed using a 5 mm side-length cube. For each measurement, marker points from ten consecutive VF oscillation cycles (approximately 190–400 frames) were manually annotated and 3D-reconstructed using in-house software (see Figure 3c). Depending on the hemilarynx, some sutures were excluded due to insufficient visibility in the video. Subsequently, the suture trajectories were used to calculate the 3D parameters.

The 3D reconstruction of the superior VF surface was based on the method described by Semmler et al. [17]. A laser grid was projected onto the VF (see Figure 3b). The 3D coordinates were reconstructed from the 2D laser dot positions in high-speed footage. The calibration was performed using in-house software routines described by Semmler et al. [18]. Ten consecutive oscillation cycles were manually annotated and reconstructed using MATLAB R2020b (The Mathworks, Inc., Natick, MA, USA) (see Figure 3d). Finally, the precise VF edge from glottis segmentation was projected onto a surface approximated through the laser points to calculate the lateral displacements along the complete VF.

#### 2.4.2. General Phonation Parameters

We assessed phonation parameters for a general evaluation and comparison with standard physiological values from other studies. These included mean subglottal pressure (*P*_sub_), sound pressure level (SPL), glottal resistance (*R*_B_) as described by Alipour et al. [20], and fundamental frequencies (*f*_0_) based on the acoustic and subglottal pressure signals. Basic descriptive statistics were used to analyze the dataset (*N* = 96) and a subset at the phonation onset flows F0 (*N* = 24).

#### 2.4.3. Three-Dimensional Parameters

The 3D parameters were derived independently from the reconstructed data of the medial and superior VF surfaces, including VF displacements and velocities. These parameters were based on the sewn-in marker points for the medial surface and the projected laser dots on the superior surface. The parameters were computed based on their mean periods. They included mean and maximum absolute velocity values (*v*_mean_ and *v*_max,_ respectively), as well as mean and maximum lateral displacements (*z*_mean_ and *z*_max_) and vertical displacements (*y*_mean_ and *y*_max_) for both datasets. The lateral displacements of the superior surface were derived from the calibrated lateral movement of the vocal fold edge and for the medial surface via the lateral portions of the mean suture trajectories. The vertical displacements of the superior surface were derived from the vertical movement of all laser points projected on the vocal fold during oscillations and for the medial surface via the vertical portion of the mean suture trajectories. The results in longitudinal directions were omitted as they were negligible and often indistinguishable from noise, comparable to previous studies [21]. For the superior VF surface, apart from validation purposes, we exclusively considered data that were eventually available in vivo from our 3D laser endoscopy system, including the glottal area waveform (GAW) based on 2D segmentation [22] and 3D laser dots, while omitting the projected suture data from a previous study [13].

#### 2.4.4. Empirical Eigenfunctions

We utilized empirical eigenfunctions (EEFs) as principal component analysis to process the medial vocal fold (VF) surface trajectories. Specifically, we selected eigenvalues (EVs) that accounted for 95% of the total oscillation energy, typically involving 2–4 EVs. We excluded the remaining 5% of the signal energy to reduce noise. EEFs allowed us to decompose VF oscillations into fundamental components [14]. The 3D parameters of the medial surface were computed based on these filtered trajectories. Using EEFs, we analyzed the energy distribution across different oscillations, examining patterns that emerged with varying flow rates, elongations, and adductions.

#### 2.4.5. Mucosal Wave

Mucosal wave propagation is characterized by the wave-like movement of the VFs, which moves perpendicular to the VF from the inferior to the superior VF surface during phonation. It can be an essential marker for evaluating VF health and diagnosing pathologies. In this study, we used it to assess the physiological behavior of our model. Additionally, by analyzing the lateral portion of the mucosal wave in human hemilarynges and 3D-reconstructed VF surfaces, we can gain valuable insights into the biomechanical properties and functional integrity of the analyzed hemilarynges.

The mucosal wave was assessed by calculating the phase delay of the sewn-in marker points on the medial VF surfaces. The phase shift was calculated from the oscillations’ mean periods of the sutures in the middle column (C3) in degrees, with respect to the bottom suture of that column (R1) (see Figure 3c). We focused on the lateral portion of the phase delay, as the bottom sutures moved mainly in the lateral direction.

#### 2.4.6. Statistical Analysis

Statistical analysis was conducted using IBM SPSS Statistics version 29.0.0.0 (241) (IBM Corporation, Armonk, NY, USA). First, Pearson correlation coefficients were computed individually for each hemilarynx. The analysis assessed the VF manipulations and 3D parameters of the superior surface against the 3D parameters of the medial surface, as well as the percentage energy values of the two highest eigenvalues (EV1 and EV2) for *p* < 0.05. Subsequently, the average of the correlation coefficients across the four hemilarynges was calculated. A linear regression analysis was performed for corresponding pairs of parameters (e.g., *v*_mean,sup_ and *v*_mean,med_) that showed significant correlations for three or four coefficients. Linear regression was chosen for data analysis since we found strong linear connections between the parameters of the superior and medial VF surfaces in the preceding study [13].

## 3. Results

### 3.1. General Phonation Parameters

**Figure 4:**
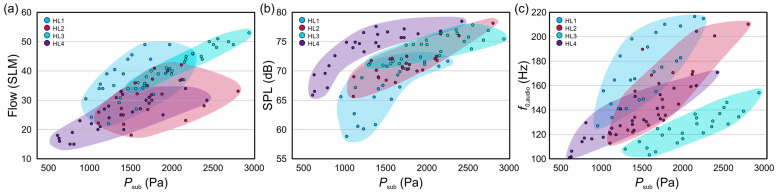
General phonation parameters of all four hemilarynges: (**a**) mean flow rate over mean subglottal pressure (*P*_sub_); (**b**) sound pressure level (SPL) over mean subglottal pressure; (**c**) fundamental frequency of the acoustic signal (*f*_0,audio_) over mean subglottal pressure.

The results of the general phonation parameters are shown in Table 2 and visualized in Figure 4. The fundamental frequency derived from the audio (*f*_0,audio_) and subglottal pressure signal (*f*_0,Psub_) showed approximately the same results, highlighting the validity of both methods. Subglottal pressures (*P*_sub_) were between 609 Pa and 2804 Pa, with the lowest values for HL4 and the highest for HL3. Values for HL1 showed a large variety in the required phonation onset flow and an exponential saturating curve for SPL. The results of HL2 are tightly clustered and require medium onset flows for phonation, with some measurements indicating low onset flows. The SPL values for HL2 showed a linear increase and significant variation in fundamental frequencies due to flow and VF adduction changes. For HL3, there was a linear increase in flow with respect to subglottal pressures, with data points closely clustered. This group exhibited the highest flow rates, high SPL, and the lowest overall frequencies. HL4 required the lowest onset flow to initiate VF oscillation, and the subglottal pressures needed for maintaining VF oscillation were low, approximately 600 Pa. At the same time, the respective SPL values were average over all measurements. For increasing *P*_sub_, the SPL showed an increase in the form of an exponential saturating curve, and overall, HL4 achieved the highest SPL values.

### 3.2. Three-Dimensional Parameter Results

**Figure 5:**
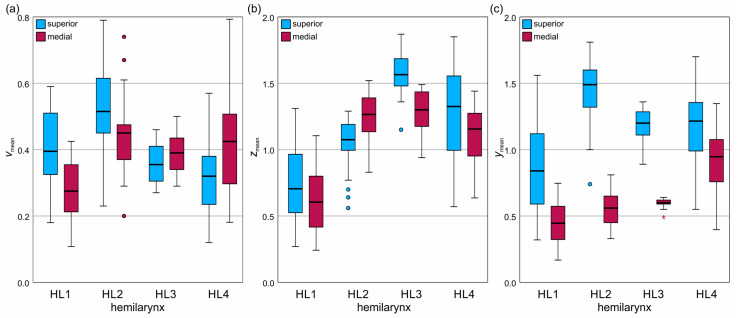
Comparison between the 3D parameters of the medial (red) and superior VF surfaces (blue) of the four analyzed hemilarynges (HL1-HL4): (**a**) mean velocity (*v*_mean_); (**b**) mean lateral displacement (*z*_mean_); (**c**) mean vertical displacement (*y*_mean_).

A comprehensive table of the evaluated 3D parameters from all hemilarynges is provided in Supplementary Material S1. Figure 5 compares the mean 3D parameters from the superior and medial VF surfaces of hemilarynges HL1 to HL4, with the mean surface velocities (*v*_mean_) in Figure 5a, mean lateral displacements (*z*_mean_) in Figure 5b, and mean vertical displacements (*y*_mean_) in Figure 5c.

Regarding the velocities, HL1 and HL2 showed higher superior values, while HL3 and HL4 showed higher medial values. HL3 had a narrower range, with overall velocities averaging 0.4 m/s and ranging from 0.1 m/s to 0.8 m/s.

In lateral displacements in Figure 5b, HL1, HL3, and HL4 have higher superior displacements, whereas HL2 shows the opposite trend. Displacement range varied significantly, with HL1 in the range of 0.6 mm–0.7 mm, HL2 in the range of 1.1 mm–1.3 mm, HL3 closely clustered in the range of 1.3 mm–1.6 mm, and HL4 in the range of 1.2 mm–1.3 mm.

Vertical displacements in Figure 5c are consistently higher on the superior surface for all hemilarynges, although the superior values for HL4 are closer to the medial values than the other hemilarynges. Ranges for the median values were between 0.8 mm and 0.4 mm for HL1, 1.6 mm and 0.6 mm for HL2, 1.2 mm and 0.6 mm for HL3, and 1.2 mm and 0.9 mm for HL4. HL1 and HL4 showed high variances, HL2 and HL3 showed high differences between superior and medial data, and HL3 showed low variation for the medial data.

### 3.3. Correlation Analysis

The correlation results between the analyzed parameters are shown in Table 3. Each cell shows the mean values of the four Pearson correlation coefficients of the four hemilarynges with the respective standard deviations. Two asterisks (**) on the values indicate that all individual correlation results are statistically significant with *p* < 0.05, and one asterisks (*) shows that at least three of the four values have significant correlation coefficients.

We found no significant correlations between the VF manipulations of elongation and adduction and the 3D parameters. Parameter pairs that correspond to superior and medial (e.g., *v*_mean,med_ and *v*_mean,sup_) values were consistently significant, with high coefficients between 0.8 and 0.95, except for *y*_max_, where only three of the four values were significant. This may be because maximum values often depend on a singular value, making it more susceptible to outliers. Expectedly, the superior parameters of the mean and maximum velocities and vertical and lateral displacements show predominantly significant correlations with their medial counterparts, with high coefficient values ranging from 0.6 to 0.95. Conversely, we found no clear correlation between medial surface parameters and the manipulations and energy distribution of the two largest eigenvalues, EV1 and EV2.

### 3.4. Regression Analysis

**Figure 6:**
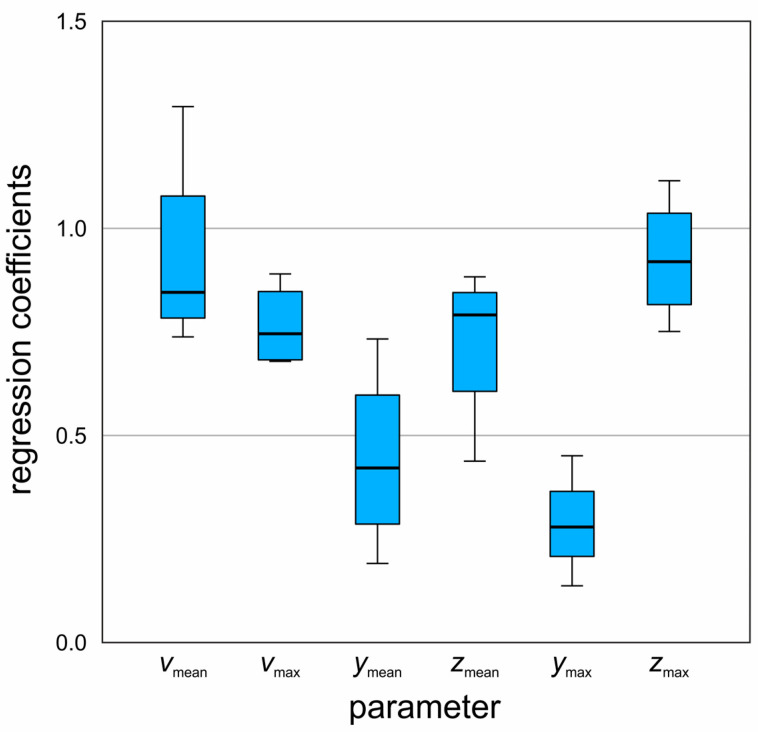
Boxplot of the regression coefficients of superior to medial 3D parameters from the four hemilarynges for mean and maximum velocities and displacements.

The results of the mean regression coefficients are shown in Table 4. The analysis of regression coefficients (B-values) from HL1 to HL4 revealed several trends and differences. For *v*_mean_, the coefficients ranged from 0.74 to 0.86 for HL1 to HL3, respectively, but significantly increased to 1.29 in HL4. The *v*_max_ values remained in a similar range across all scenarios, between 0.68 and 0.90 (0.22 variation). The parameter *y*_mean_ showed a large variety, with coefficients ranging from 0.19 to 0.46 from HL1 to HL3, respectively, and a notable rise to 0.73 in HL4, indicating an increase from 0.27 to 0.54 when HL4 was included. *z*_mean_ was stable between 0.81 and 0.88 for HL1 and HL3, respectively, but HL4 deviated with a coefficient of 0.44, marking an increase from 0.07 to 0.44 when HL4 was considered. For *y*_max_, coefficients ranged between 0.14 and 0.45 (0.31 variation), excluding the result for HL3 due to a *p*-value > 0.05. Finally, *z*_max_ coefficients varied from 0.75 to 1.12, showing a 0.37 difference from HL1 to HL4, respectively. Additionally, these results are visualized in Figure 6. HL4 often showed significant deviations compared to values ranging from HL1 to HL3, particularly for *v*_mean_, *y*_mean_, and *z*_mean_. These results mostly align with the expectations, showing predominantly lower 3D parameter values on the medial surfaces compared to the superior surfaces. Notably, maximum values for the vertical displacements were significantly higher on the superior surface. Mean lateral displacements, *z*_mean_, were also lower medially, although this difference was less pronounced. For *z*_max_, medial surface values were slightly lower in most cases than those on the superior surface, with one instance showing a higher medial value. Overall, more extensive and faster movements were observed on the superior surface, consistent with the expected behavior.

### 3.5. Empirical Eigenfunctions

**Figure 7:**
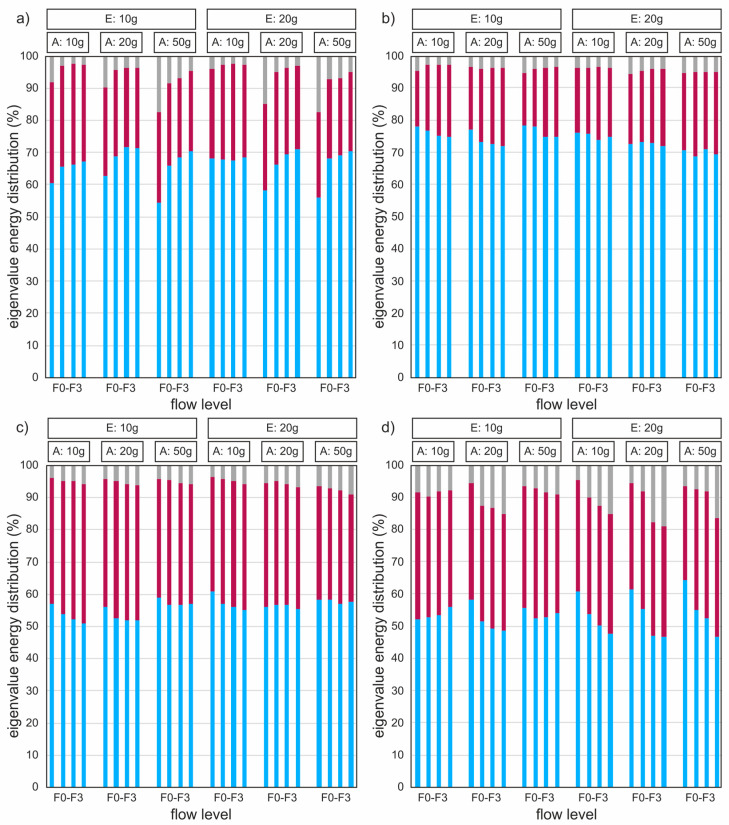
Percentage energy distribution of the eigenvalues, computed from the medial VF surface trajectories of the four hemilarynges with (**a**) HL1; (**b**) HL2; (**c**) HL3; (**d**) HL4. The first EEF is blue, the second is red, and residual eigenvalues are gray. The elongation (E) and adduction (A) weights applied to the hemilarynx are shown above the graphs. Each setting was tested at four increasing flow levels, starting with the onset flow (F0) and three incremental 5 SLM steps (F1–F3).

The percentage energy distribution of the medial vocal fold (VF) surface trajectories of the four hemilarynges is illustrated in Figure 7. The magnitudes of the first eigenvalues (EVs) are depicted in blue, the second in red, and the remaining in gray. Above the graphs, the elongation (E) and adduction (A) weights applied to the hemilarynx during the measurement are indicated. For each setting, the hemilarynges were tested at four increasing flow levels, starting with the onset flow (F0) and followed by three incremental five SLM steps.

The first EV was generally responsible for the overall mediolateral movement and the convergent–divergent shape of the VF, accounting for averages ranging from 53% to 74% of the total oscillation energy of the four hemilarynges. The second EV primarily involved the middle and superior sutures displacing near the VF edge. This introduced the mucosal wave moving from inferior to superior, as shown in File S2 in the Supplementary Materials. On average, the second EV contained between 29% and 68% of the energy of the first EV. The third EV added complexity to the simpler circular trajectory, transforming it into more intricate shapes. Although the third EV’s contribution to the overall oscillation was smaller, it captured more subtle details that defined the trajectory’s unique characteristics.

The number of remaining (gray) EVs mainly depended on the overall oscillations’ complexity and amplitudes. The more complex the oscillations, the more EVs were needed to describe them. Additionally, since noise from manual annotation was independent of the oscillation amplitudes and constant across all experiments, this resulted in lower signal-to-noise ratios for lower amplitudes. Consequently, the first and second EVs contain less energy in these cases, such as HL1 at onset flows (F0). These effects occur simultaneously and cannot always be easily distinguished.

There was no apparent influence of elongation or adduction on the energy distribution in all cases. For HL1, the first EV at F0 had the lowest energy, and with more flow, there was more energy in EV1. For HL2 and HL3, the first EV tended to decrease with the increased flow. For HL4, there was an overall strong downward trend in EV1 with increased flow rates, except in the case of E10/A10, which was reversed. HL2 had the highest EV1 energies, while HL4 had the lowest EV1 energies and the highest for HL3 and residuals (10%). However, this was expected, as HL4 had the most complex oscillations due to a subharmonic component in the signal. In HL4, subharmonic oscillations were present in the first EV in nine cases, causing a more complex oscillation and low values for EV1, particularly in cases where EV1 was below 50%.

A general finding is that subharmonics eventually appear when the EVs are analyzed individually. The higher the EV at which they appear (e.g., EV3 or higher), the more stable and uniform the resulting VF oscillation. For HL2 and HL3, the first subharmonic EVs appeared in EV3, while for HL1, they consistently first appeared at higher EVs. Only HL4 had subharmonics before EV2 and EV3. Despite their effect on the energy distribution and some distortion in the visual representation of the mean period, these subharmonics had little or no impact on the general phonation parameters, 3D parameter calculation, or mucosal wave propagation.

### 3.6. Mucosal Wave

**Figure 8:**
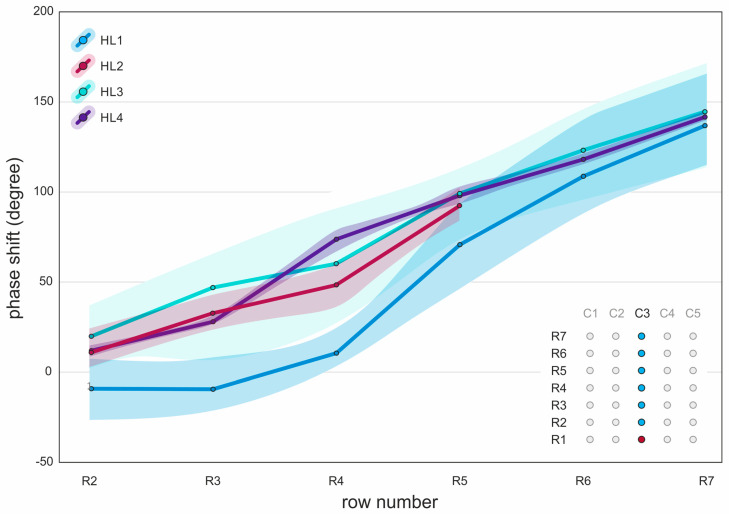
Mean lateral phase shift of sutures on the medial surface from the middle column (C3) in relation to the bottom suture (R1) of column C3. Lines represent the mean phase shift values for each hemilarynx (HL1, HL2, HL3, and HL4). Shaded areas around the lines indicate the respective minimum to maximum ranges. Measurements are plotted against row numbers (R2 to R7).

The results of the mucosal wave propagation, illustrated in the form of a mean lateral phase shift from the middle column sutures (C3) in relation to the bottom suture in row 1 (R1), are shown in Figure 8. The data indicate a clear mucosal wave propagation both visually and from the suture data for all measurements. A primarily linear increase in the phase shift was observed from the bottom to the top, with maximum values for R7 ranging from approximately 120° to 170°. HL1 started with a flat curve, but ended with similar results as the other hemilarynges. HL2 is missing two top-row sutures due to insufficient visibility, but shows a similar general direction in the phase shift. HL3 exhibited a large range of phase shift values, while HL4 demonstrated a very narrow range. Increasing flow rates, adduction, and elongation had no clear influence on mucosal wave propagation.

## 4. Discussion

In this study, we synchronously recorded the medial and superior VF surfaces of four human ex vivo hemilarynges using two high-speed cameras, reconstructing 3D coordinates under various conditions. We expanded our previous work [13] to a comprehensive dataset of 96 individual experiments aimed at investigating the relationships in the oscillatory behavior between these surfaces. This contributes to a deeper understanding of VF dynamics, enhances the application of 3D laryngoscopy in voice research, and provides data that enable advanced laryngeal modeling.

However, our goals require that the oscillation behavior of the ex vivo larynges closely approximate in vivo VF oscillations. Despite some experimental constraints, our results show that the hemilarynges exhibit near-physiological oscillatory behavior. For instance, the vocal folds (VFs) did not contact the glass plate to avoid contamination and obstructing the view. This lack of contact meant that VF collision was not replicated, making the oscillation more similar to someone with glottis closure insufficiency. However, this did not seem to affect the VF oscillations significantly, as our parameters align well overall with full larynx studies, and complex mucosal wave propagations were present in all experiments. A shortcoming of this study is the exclusive analysis of male larynges due to limited availability, as the physiology of female VFs differs in certain aspects from male VFs. In future studies, the results of this work need to be complemented by further experiments on a variety of cases, including female larynges and pathologies.

In contrast to general phonation parameters, such as fundamental frequencies and SPL, it is challenging to effectively compare the results of different studies regarding 3D data due to significant differences in the methods of data acquisition and subsequent processing, as well as the individual biological properties of the larynges for both in vivo and ex vivo studies. However, some trends and commonalities in the results can be identified when a variety of different outcomes are considered.

Firstly, our general phonation parameters aligned well with those of Semmler et al., who performed an in vivo study using a 3D laryngoscopy system in 10 subjects [9]. We found that our measured lateral displacements of approximately 1 mm were overall similar. However, their vertical displacements were consistently higher, a pattern observed only in our maximum values. However, their maximum velocities were higher at 0.99 m/s to 2.3 m/s compared to our average value of 0.86 m/s. Furthermore, our data align well with the findings from another in vivo study by George et al. [12], who reported maximum VF velocities ranging from 0.63 m/s to 0.92 m/s for VF opening, compared to our average of 0.86 m/s. Our maximum vertical displacements were higher, averaging 2.49 mm, compared to their values, ranging from 0.7 mm to 1.2 mm. However, our maximum lateral displacements are in the same range, with our values averaging at 1.48 mm and their values ranging from 1.2 mm to 1.6 mm. Another in vivo study by Larsson and Hertegard investigating seven subjects (three male and four female) found maximum lateral displacements between 1 mm and 2 mm, maximum vertical displacements ranging from 0.5 mm to 2 mm, and maximum velocities ranging from 0.38 m/s to 1.3 m/s [23]. These findings closely align with our results of 1.47 mm for lateral displacement, 2.49 mm for vertical displacements, and 0.86 m/s for velocity, although our vertical displacements are slightly higher. A recent study by Patel et al. investigated 23 healthy adult subjects during sustained phonation using 3D laryngoscopy [24]. Comparing their male subjects at normal pitch and loudness with our results, we found similar average frequencies (124 Hz vs. 145 Hz) and SPL (73 dB vs. 72 dB). Our average superior surface velocity of 0.4 m/s aligns well with their reported mean opening velocity in vertical directions of 0.42 m/s but less with their closing velocity of 0.7 m/s. Our overall maximum velocity of 0.86 m/s is close to their value of 1.02 m/s for mean opening velocity but considerably lower than their mean closing velocity of 1.56 m/s. However, our maximum displacements are higher (1.47 mm lateral and 2.49 mm vertical) than theirs (0.8 mm and 1.5 mm, respectively). This discrepancy most likely results from different methods of data collection and different parameter calculation methods. Despite this, the trend of vertical amplitudes being over 50% higher is consistent with our data, indicating a good agreement in the general oscillation behavior between our ex vivo data and reported in vivo studies. This general trend that our 3D parameters are mostly consistent with the literature equally applies to ex vivo studies, both in human hemilarynges [16,25,26], full larynx [27], and animal studies [28].

Furthermore, we utilized empirical eigenfunctions (EEFs) primarily to reduce noise in our data and to analyze the vibratory patterns of the VFs. Our results align with the existing literature, indicating that two eigenvalues (EVs) are often sufficient to explain the majority of the oscillatory energy [21]. The first EV accounted for approximately 53% to 74% of the total oscillation energy, representing the overall mediolateral movement of the sutures from rows R3 to R5 and transition to a vertical movement above R5. This results in the previously reported convergent–divergent VF shape by Döllinger et al. [21]. The second EV primarily adds the respective vertical and lateral components to the sutures near the VF edge and contains significantly less energy (22–39%). However, Döllinger et al. observed mostly a lateral movement for EV2 when examining the EVs individually, possibly due to differences in the investigated VF surface area. When considering the combination of EV1 and EV2, we found that introducing EV2 led to the distinct mucosal wave movement from inferior to superior, as shown in Supplementary File S2. While contributing less to the overall energy, the third EV captured more subtle details of the trajectories and may be useful when combined with other 3D parameters for a more comprehensive analysis. Notably, HL4 exhibited strong subharmonics in the first EV in nine of 24 experiments. Subharmonics are supposed to adversely affect the acoustic signal, creating a rough voice in running speech [29]. Regarding VF dynamics, subharmonic oscillation in the time domain is characterized by periodic fluctuations in the amplitude of the VFs. The simplest and most common form was a superimposition of an oscillation at half the fundamental frequency, alternating a high with a low VF amplitude [30]. However, depending on the spectral energy distribution and the number of subharmonic oscillations, these can also produce more complex oscillation patterns.

However, except for lowering the energy in the first EV to below 50% in most cases, this did not adversely affect general phonation parameters or 3D parameters, indicating stable oscillations despite added complexity in the signal. This finding is similar to Omori et al., who often found normal values for shimmer and jitter, despite subjects having rough voices and subharmonics in the voice spectrum [31]. Using EEFs is particularly advantageous for reducing noise and data dimensionality (i.e., reducing complexity), which may benefit training advanced computer models. For instance, Henningson et al., who applied the M5 geometry from Scherer et al. to their soft tissue deformation model, could benefit from adopting a more realistic VF shape and trajectories from our hemilarynx data, especially by calculating averaged VF trajectories that are reduced in dimensionality using EEFs [32]. Likewise, other kinematic and numerical models could equally benefit from our more realistic 3D dataset reflecting a wide range of VF dynamics [33,34]. AI models, such as those of Gomez et al. that only used training data from synthetic vocal fold models, could also gain added value from using our 3D data [35].

Our analysis also revealed pronounced mucosal wave propagation from the inferior to superior region across all hemilarynges, manifesting as a linear increase in lateral phase delay. For most cases, lateral phase delays between row one (most inferior) and row seven (most superior) ranged from approximately 120° to 180°. These findings align with Döllinger et al., who found similar lateral phase delays in a similar range in a canine in vivo model [36], as well as Boessenecker et al., who reported a lateral phase delay of approximately 180° from inferior to superior [25] and Titze et al. stating values between 30° and 60° per mm in an animal model [37]. Our statistical analysis yielded no consistent correlations between mucosal wave propagation and the superior 3D parameters and the manipulations of flow, adduction, and elongation. This suggests that it may not be possible to predict mucosal wave propagation on the medial surface based on these parameters and manipulations.

Regarding predicting medial surface behavior by observing the superior surface, we found expected high correlations between the 3D parameters of the superior and medial surfaces of the VF [13]. We employed linear regression to quantify these correlations further, building on previous work that indicated strong linear connections in HL1. We examined corresponding parameter pairs, such as displacements on the superior surface versus displacements on the medial surface, to identify candidates that exhibited consistent behavior across all larynges. Although there was some variability across the individual larynges, the differences between the superior values and their medial counterparts were in a narrow range for certain parameters. Specifically, the maximum velocity and vertical displacement showed narrow ranges (0.6–0.8 m/s for *v*_max_ and 0.2–0.4 mm for *y*_max_, respectively). The range of *v*_max_ is particularly noteworthy for clinical applications, as VF collision speeds are associated with polyp generation and phono-trauma. Thus, *v*_max_ is our most promising parameter for predicting medial surface behavior and assessing the risk of VF damage.

The next step to substantiate these connections would be validation and comparison with in vivo data. This, however, presents a challenge as there is currently no method to directly analyze the properties of the medial VF surface during an in vivo examination. The observed linear relationships indicate that superior surface data may suffice for evaluating collision speeds and predicting future damage due to high collision forces. This implies that detailed medial VF surface information might not be necessary for this specific parameter, highlighting the importance of establishing guidelines for identifying potentially harmful VF velocities. Nevertheless, absolute velocity measurements are needed to investigate the correlation between VF trauma and VF velocity further. Three-dimensional laryngoscopy, which provides absolute values, could be particularly valuable. This technology could play a crucial role in identifying patients at risk and preventing VF-related diseases. Future research should focus on these guidelines and explore threshold velocities associated with potential VF damage.

Additionally, the hypothesis that superior VF data are sufficient could be tested through targeted studies. By examining the correlations between superior VF speeds and the emergence of VF trauma, we can further substantiate our prediction that the connection is mostly linear. Despite the challenges, validating our results with in vivo data would further support the development of these critical clinical tools and the implementation of 3D laryngoscopy as a preventive measure in clinical practice.

Overall, our comprehensive dataset and analyses contribute to a deeper understanding of VF dynamics, paving the way for enhanced 3D laryngoscopy applications and advanced laryngeal modeling in voice research.

## 5. Conclusions

This study systematically examined four hemilarynges in 24 different measurement conditions by 3D reconstructing and analyzing the superior and medial vocal fold (VF) surfaces. Comparing general phonation parameters, such as fundamental frequencies and subglottal pressures, as well as mucosal wave propagation, showed comparable results with both in vivo and ex vivo studies. Although each hemilarynx was comprehensively analyzed, the small number of hemilarynges is a limitation of this work. Future studies will build on this and expand the dataset.

The computed 3D surface parameters of mean and maximum velocities and displacements were used to investigate the relationships of the oscillatory behavior between the superior and medial VF surfaces. Ultimately, these findings can be used to predict the properties of the in vivo hidden medial VF surface based on the data obtained from the superior surface using a 3D laryngoscope. The linear relationships between the 3D parameters of the medial and superior surfaces shown in the previous study were also observed in the three additional hemilarynges in this work. This demonstrates that interindividual differences are likely not an obstacle to valid correlations. The regressions further showed some commonalities across all hemilarynges, especially for the maximum values of the VF velocities and vertical displacements. This is a valuable finding when considering that the vocal folds’ closing velocity and contact force are indicators of polyp formation and phono-trauma. This shows that 3D laryngoscopy, by providing absolute values for velocities and displacements compared to the relative values of 2D laryngoscopy, can provide valuable information for ENT diagnostics and potentially be used for disease prevention. However, more research on critical velocity thresholds is needed before implementing this in a clinical setting.

Finally, another outcome of this study is the open source dataset of 96 individual experiments of ten oscillation cycles each. This contains the synchronous 3D data of the superior surface and the trajectories of the medial surface, as well as the general phonation parameters. This provides other research groups without access to realistic human vocal fold data with a broad range of resources for various computer modeling applications, such as AI or numerical modeling.

## Figures and Tables

**Table 1:** Hemilarynges used in this study and essential information on body donors.

ID	Sex	Age (Years)	Height (cm)	Weight (kg)
HL1	Male	81	165	52.1
HL2	Male	90	185	68
HL3	Male	73	168	54.4
HL4	Male	76	163	63.5

**Table 2:** Range of general phonation parameters of all four hemilarynges for phonation onset and over all recordings: fundamental frequencies, mean subglottal pressure (*P*_sub_), flow rate, glottal resistance (*R*_B_), and sound pressure level (SPL).

Parameter (unit)	*f*_0,audio_ (Hz)	*f*_0,*P*sub_ (Hz)	*P*_sub_ (Pa)	Flow Rate (SLM)	*R*_B_ (Pa/SLM)	SPL (dB)
Phonation onset (*n* = 24)	139 ± 21	140 ± 21	1149 ± 808	26 ± 8	49 ± 9	68 ± 6
All recordings (*n* = 96)	145 ± 19	145 ± 19	1657 ± 293	34 ± 9	50 ± 6	72 ± 3
Minimum values	101	102	609	15	31	59
Maximum values	216	217	2804	53	94	78

**Table 3:** Mean values and standard deviations of the Pearson correlation coefficients of the four hemilarynges analyzed. ** Significant for all 4 HLs; * significant for 3 out of 4 HLs.

	*v*_mean,med_	*v*_max,med_	*y*_mean,med_	*z*_mean,med_	*y*_max,med_	*z*_max,med_	EV1	EV2
Elongation	0.02 ± 0.23	0.15 ± 0.23	0.06 ± 0.17	−0.14 ± 0.39	0.03 ± 0.22	−0.16 ± 0.39	0.013 ± 0.43	−0.27 ± 0.48 **
Adduction	0.16 ± 0.32	0.07 ± 0.27	−0.14 ± 0.33	−0.34 ± 0.19	−0.01 ± 0.34	−0.25 ± 0.20	0.1 ± 0.31	−0.1 ± 0.37
flow	0.80 ± 0.17 **	0.81 ± 0.17 **	0.70 ± 0.14 **	0.65 ± 0.14 **	0.69 ± 0.13 **	0.68 ± 0.15 **	−0.57 ± 0.17 *	0.27 ± 0.18
*v*_mean,sup_	0.95 ± 0.03 **	0.90 ± 0.08 **	0.84 ± 0.17 **	0.70 ± 0.21 *	0.87 ± 0.11 **	0.75 ± 0.23 *	−0.57 ± 0.14 *	0.35 ± 0.29
*v*_max,sup_	0.94 ± 0.03 **	0.91 ± 0.07 **	0.79 ± 0.24 *	0.71 ± 0.19 *	0.83 ± 0.14 **	0.76 ± 0.20 **	−0.56 ± 0.1 **	0.31 ± 0.3
*y*_mean,sup_	0.79 ± 0.12 **	0.69 ± 0.15 **	0.85 ± 0.10 **	0.93 ± 0.05 **	0.84 ± 0.10 **	0.92 ± 0.05 **	−0.46 ± 0.16	0.51 ± 0.17
*z*_mean,sup_	0.74 ± 0.10 **	0.59 ± 0.23 *	0.76 ± 0.16 **	0.82 ± 0.12 **	0.80 ± 0.11 **	0.83 ± 0.13 **	−0.27 ± 0.22	0.5 ± 0.12 **
*y*_max,sup_	0.63 ± 0.16 **	0.55 ± 0.25 *	0.56 ± 0.28 *	0.68 ± 0.06 **	0.60 ± 0.16 *	0.71 ± 0.08 **	−0.45 ± 0.3	0.52 ± 0.2
*z*_max,sup_	0.69 ± 0.14 **	0.52 ± 0.22	0.73 ± 0.20 **	0.92 ± 0.07 **	0.74 ± 0.17 **	0.92 ± 0.08 **	−0.35 ± 0.23	0.58 ± 0.14 **

**Table 4:** Regression coefficients of the four hemilarynges, analyzed with the superior 3D parameters as independent variables and the medial surface parameters as dependent variables.

Hemilarynx ID	Three-Dimensional Parameter	B (Medial var.)	B (const.)	Std. Error	T-Value	*p*-Value	*R*^2^ (Medial var.)
HL1	*v*_mean,sup_	0.74	−0.02	0.02	32.0	0.001	0.98
	*v*_max,sup_	0.68	0.01	0.03	20.9	0.001	0.95
	*y*_mean,sup_	0.46	0.04	0.02	26.3	0.001	0.97
	*z*_mean,sup_	0.88	−0.01	0.03	26.8	0.001	0.97
	*y*_max,sup_	0.45	0.14	0.07	6.5	0.001	0.41
	*z*_max,sup_	1.12	0.04	0.05	22.9	0.001	0.90
HL2	*v*_mean,sup_	0.86	−0.01	0.08	11.6	0.001	0.85
	*v*_max,sup_	0.81	−0.03	0.08	10.2	0.001	0.82
	*y*_mean,sup_	0.38	0.01	0.06	6.3	0.001	0.63
	*z*_mean,sup_	0.78	0.44	0.11	6.9	0.001	0.67
	*y*_max,sup_	0.14	0.43	0.03	4.1	0.001	0.41
	*z*_max,sup_	0.88	0.64	0.1	14.8	0.001	0.90
HL3	*v*_mean,sup_	0.83	0.09	0.08	11.1	<0.001	0.84
	*v*_max,sup_	0.69	0.12	0.04	16.7	<0.001	0.92
	*y*_mean,sup_	0.19	0.37	0.04	4.7	<0.001	0.48
	*z*_mean,sup_	0.81	0.02	0.11	7.2	<0.001	0.69
	*y*_max,sup_	0.10	1.46	0.05	1.8	0.085	0.09
	*z*_max,sup_	0.75	0.79	0.04	18.9	<0.001	0.94
HL4	*v*_mean,sup_	1.29	0.02	0.09	14.4	0.001	0.90
	*v*_max,sup_	0.90	0.21	0.15	6.1	0.001	0.61
	*y*_mean,sup_	0.73	0.06	0.08	9.3	0.001	0.79
	*z*_mean,sup_	0.44	0.54	0.11	4.0	0.001	0.40
	*y*_max,sup_	0.28	0.78	0.09	3.2	0.001	0.28
	*z*_max,sup_	0.96	0.48	0.16	6.0	0.001	0.61

## Data Availability

The original data presented in the study is openly available on figshare, at http://doi.org/10.6084/m9.figshare.27118785.

## Supplementary Materials

The following supporting information can be downloaded at: https://www.mdpi.com/article/10.3390/bioengineering11100977/s1, Tables in S1: hemilarynx 3D parameters.docx, containing four tables with the 3D parameter data of hemilarynges HL1, HL2, HL3 and HL4; File S2: eigenvalues and medial vocal fold trajectories.mp4.
