# Supplementary material for: Three-Dimensional Analysis of Vocal Fold Oscillations: Correlating Superior and Medial Surface Dynamics Using Ex Vivo Human Hemilarynges
Source: Bioengineering (Basel). 2024 Sep 28;11(10):977. doi: 10.3390/bioengineering11100977 (PMC11505270; doi:10.3390/bioengineering11100977)
Supplement: Supplementary file 1 [file bioengineering-11-00977-s001.zip › S1 Hemilarynx 3D parameters.pdf]

S1: 3D vocal fold surface parameters computed for HL1 to HL4

Table S1 3D parameters of Hemilarynx 1 (HL1), calculated from the reconstructed 3D data of the superior and medial vocal fold surface. The flow rate F0 always corresponds to the individual phonation onset flow of the respective elongation and adduction setting, with three step-wise 5 slm increses (e.g., F3 is the onset flow plus 15 slm).

elongation (g)	adduction (g)	flow rate (a.u.)	$v_{mean,sup}$ (m/s)	$v_{max,sup}$ (m/s)	$y_{mean,sup}$ (mm)	$y_{max,sup}$ (mm)	$z_{mean,sup}$ (mm)	$z_{max,sup}$ (mm)	$v_{mean,med}$ (m/s)	$v_{max,med}$ (m/s)	$y_{mean,med}$ (mm)	$y_{max,med}$ (mm)	$z_{mean,med}$ (mm)	$z_{max,med}$ (mm)
10	10	F0	0.22	0.38	0.59	1.21	0.55	0.70	0.15	0.26	0.33	0.71	0.46	0.78
10	10	F1	0.40	0.63	1.14	2.04	0.95	1.24	0.28	0.44	0.55	1.18	0.83	1.35
10	10	F2	0.51	0.77	1.35	2.55	1.14	1.46	0.37	0.59	0.60	1.65	1.02	1.63
10	10	F3	0.59	0.99	1.56	2.83	1.31	1.58	0.43	0.74	0.75	1.58	1.11	1.76
10	20	F0	0.22	0.34	0.42	0.79	0.40	0.51	0.14	0.26	0.23	0.52	0.35	0.54
10	20	F1	0.38	0.65	0.83	1.71	0.71	0.84	0.26	0.41	0.44	0.95	0.62	0.96
10	20	F2	0.51	0.80	1.02	1.84	0.89	1.04	0.34	0.54	0.55	1.22	0.72	1.22
10	20	F3	0.52	0.88	1.11	2.10	0.98	1.17	0.38	0.60	0.59	1.21	0.76	1.32
10	50	F0	0.18	0.33	0.36	1.20	0.27	0.35	0.13	0.24	0.18	0.41	0.25	0.45
10	50	F1	0.31	0.47	0.57	1.57	0.51	0.60	0.21	0.35	0.27	0.68	0.40	0.70
10	50	F2	0.39	0.62	0.77	1.89	0.66	0.79	0.28	0.44	0.38	0.85	0.51	0.94
10	50	F3	0.49	0.89	0.98	3.60	0.75	0.91	0.37	0.61	0.50	1.10	0.64	1.20
20	10	F0	0.34	0.55	0.85	1.80	0.70	0.90	0.22	0.34	0.41	0.91	0.59	0.95
20	10	F1	0.43	0.70	1.10	1.86	0.80	0.95	0.28	0.43	0.52	1.11	0.77	1.16
20	10	F2	0.53	0.83	1.31	2.16	1.04	1.26	0.34	0.55	0.63	1.35	0.93	1.41
20	10	F3	0.59	1.01	1.43	2.44	1.12	1.37	0.40	0.63	0.68	1.41	1.02	1.57
20	20	F0	0.18	0.34	0.36	0.89	0.31	0.39	0.11	0.22	0.19	0.41	0.26	0.46
20	20	F1	0.36	0.58	0.81	1.71	0.61	0.74	0.24	0.37	0.38	0.83	0.57	0.92
20	20	F2	0.45	0.79	1.00	2.06	0.73	0.87	0.32	0.52	0.50	1.09	0.74	1.21
20	20	F3	0.54	0.90	1.13	2.25	0.99	1.17	0.39	0.67	0.62	1.28	0.86	1.39
20	50	F0	0.19	0.31	0.32	0.90	0.30	0.38	0.12	0.24	0.17	0.41	0.24	0.41
20	50	F1	0.34	0.55	0.59	1.45	0.49	0.72	0.22	0.35	0.32	0.72	0.42	0.68
20	50	F2	0.38	0.56	0.63	1.57	0.54	0.65	0.25	0.40	0.35	0.78	0.42	0.74
20	50	F3	0.47	0.76	0.81	1.77	0.64	0.77	0.32	0.55	0.46	1.06	0.53	1.00

Table S2 3D parameters of Hemilarynx 2 (HL2), calculated from the reconstructed 3D data of the superior and medial vocal fold surface. The flow rate F0 always corresponds to the individual phonation onset flow of the respective elongation and adduction setting, with three step-wise 5 slm increses (e.g., F3 is the onset flow plus 15 slm).

elongation (g)	adduction (g)	flow rate (a.u.)	$v_{mean,sup}$ (m/s)	$v_{max,sup}$ (m/s)	$y_{mean,sup}$ (mm)	$y_{max,sup}$ (mm)	$z_{mean,sup}$ (mm)	$z_{max,sup}$ (mm)	$v_{mean,med}$ (m/s)	$v_{max,med}$ (m/s)	$y_{mean,med}$ (mm)	$y_{max,med}$ (mm)	$z_{mean,med}$ (mm)	$z_{max,med}$ (mm)
10	10	F0	0.23	0.51	1.00	2.01	0.56	0.73	0.20	0.35	0.33	0.54	0.83	1.22
10	10	F1	0.42	0.87	1.38	3.35	1.01	1.28	0.33	0.57	0.44	0.71	1.22	1.69
10	10	F2	0.46	0.99	1.39	3.10	1.04	1.37	0.35	0.62	0.46	0.68	1.27	1.78
10	10	F3	0.47	0.97	1.52	3.10	1.07	1.44	0.37	0.66	0.46	0.71	1.33	1.82
10	20	F0	0.47	1.06	1.41	2.78	1.07	1.39	0.40	0.67	0.55	0.91	1.26	1.86
10	20	F1	0.52	1.19	1.57	3.12	1.19	1.50	0.47	0.96	0.66	0.95	1.42	1.96
10	20	F2	0.51	1.10	1.60	2.94	1.22	1.45	0.45	0.92	0.65	0.90	1.42	1.97
10	20	F3	0.62	1.35	1.73	3.27	1.19	1.55	0.50	1.10	0.69	0.97	1.52	2.07
10	50	F0	0.32	0.61	0.74	1.35	0.64	0.80	0.33	0.58	0.39	0.64	0.84	1.32
10	50	F1	0.51	0.97	1.15	1.97	0.99	1.19	0.41	0.69	0.44	0.73	1.04	1.61
10	50	F2	0.56	1.14	1.42	2.28	1.10	1.35	0.48	0.82	0.55	0.84	1.23	1.79
10	50	F3	0.56	1.18	1.37	2.25	1.22	1.36	0.46	0.84	0.59	0.91	1.21	1.78
20	10	F0	0.37	0.76	1.20	2.42	0.77	1.10	0.29	0.53	0.39	0.74	1.08	1.59
20	10	F1	0.48	1.02	1.51	3.18	1.08	1.31	0.37	0.74	0.55	0.76	1.33	1.82
20	10	F2	0.53	1.06	1.69	3.91	1.04	1.42	0.43	0.90	0.61	0.87	1.44	1.95
20	10	F3	0.62	1.37	1.81	3.71	1.13	1.53	0.47	0.96	0.65	0.87	1.48	1.99
20	20	F0	0.44	0.87	1.27	3.22	0.85	1.07	0.37	0.70	0.44	0.67	1.05	1.61
20	20	F1	0.54	1.15	1.47	3.29	1.00	1.18	0.46	0.87	0.57	0.89	1.19	1.73
20	20	F2	0.61	1.30	1.60	3.62	1.15	1.41	0.45	0.97	0.62	0.93	1.35	1.90
20	20	F3	0.65	1.49	1.63	3.69	1.12	1.41	0.50	1.09	0.64	0.93	1.40	1.96
20	50	F0	0.42	0.81	1.03	2.50	0.70	0.85	0.46	0.94	0.51	0.82	1.03	1.57
20	50	F1	0.65	1.32	1.55	3.77	1.26	1.36	0.61	1.23	0.69	1.05	1.20	1.80
20	50	F2	0.72	1.60	1.59	3.54	1.29	1.42	0.67	1.40	0.76	1.15	1.31	1.91
20	50	F3	0.79	1.85	1.74	4.85	1.22	1.48	0.74	1.47	0.81	1.20	1.38	2.01

Table S3 3D parameters of Hemilarynx 3 (HL3), calculated from the reconstructed 3D data of the superior and medial vocal fold surface. The flow rate F0 always corresponds to the individual phonation onset flow of the respective elongation and adduction setting, with three step-wise 5 slm increses (e.g., F3 is the onset flow plus 15 slm).

elongation (g)	adduction (g)	flow rate (a.u.)	$v_{mean,sup}$ (m/s)	$v_{max,sup}$ (m/s)	$y_{mean,sup}$ (mm)	$y_{max,sup}$ (mm)	$z_{mean,sup}$ (mm)	$z_{max,sup}$ (mm)	$v_{mean,med}$ (m/s)	$v_{max,med}$ (m/s)	$y_{mean,med}$ (mm)	$y_{max,med}$ (mm)	$z_{mean,med}$ (mm)	$z_{max,med}$ (mm)
10	10	F0	0.27	0.64	1.13	2.10	1.59	2.31	0.32	0.55	0.60	1.66	1.31	2.40
10	10	F1	0.33	0.74	1.22	2.21	1.48	2.47	0.38	0.65	0.61	1.64	1.42	2.61
10	10	F2	0.36	0.85	1.25	2.31	1.71	2.46	0.41	0.70	0.59	1.66	1.46	2.65
10	10	F3	0.42	1.01	1.34	3.63	1.53	2.58	0.44	0.77	0.58	1.66	1.48	2.75
10	20	F0	0.28	0.68	1.11	1.99	1.61	2.32	0.33	0.56	0.59	1.64	1.32	2.44
10	20	F1	0.34	0.75	1.26	2.82	1.66	2.28	0.39	0.66	0.59	1.72	1.42	2.59
10	20	F2	0.40	0.96	1.34	3.34	1.73	2.41	0.44	0.73	0.60	1.76	1.46	2.64
10	20	F3	0.43	1.08	1.35	2.90	1.80	2.52	0.47	0.83	0.60	1.77	1.49	2.74
10	50	F0	0.27	0.62	1.04	2.06	1.55	2.14	0.32	0.54	0.56	1.51	1.22	2.30
10	50	F1	0.34	0.73	1.21	2.35	1.78	2.37	0.39	0.66	0.60	1.76	1.38	2.56
10	50	F2	0.39	0.87	1.31	2.33	1.81	2.38	0.44	0.75	0.62	1.84	1.45	2.62
10	50	F3	0.44	1.08	1.36	2.50	1.87	2.53	0.50	0.89	0.63	1.88	1.48	2.70
20	10	F0	0.28	0.59	1.11	2.07	1.47	1.94	0.31	0.52	0.62	1.63	1.18	2.28
20	10	F1	0.33	0.70	1.23	2.34	1.51	1.99	0.37	0.63	0.63	1.77	1.28	2.35
20	10	F2	0.46	0.87	1.17	1.85	1.60	2.14	0.42	0.71	0.64	1.76	1.31	2.38
20	10	F3	0.43	0.94	1.33	2.21	1.48	2.11	0.43	0.76	0.64	1.75	1.29	2.34
20	20	F0	0.27	0.66	0.97	2.24	1.36	1.80	0.30	0.54	0.55	1.51	1.06	2.13
20	20	F1	0.33	0.74	1.12	2.12	1.49	1.91	0.35	0.64	0.59	1.69	1.17	2.28
20	20	F2	0.38	0.85	1.18	2.19	1.58	1.97	0.39	0.70	0.60	1.68	1.19	2.27
20	20	F3	0.40	0.92	1.25	2.16	1.59	2.00	0.42	0.79	0.63	1.72	1.20	2.31
20	50	F0	0.28	0.69	0.89	1.73	1.15	1.57	0.29	0.54	0.49	1.33	0.94	1.91
20	50	F1	0.35	0.80	1.05	2.22	1.39	1.74	0.35	0.65	0.55	1.58	1.05	2.10
20	50	F2	0.40	0.90	1.09	2.20	1.41	1.78	0.41	0.78	0.59	1.70	1.11	2.18
20	50	F3	0.46	1.07	1.19	2.30	1.52	1.87	0.45	0.85	0.62	1.76	1.14	2.22

Table S4 3D parameters of Hemilarynx 4 (HL4), calculated from the reconstructed 3D data of the superior and medial vocal fold surface. The flow rate F0 always corresponds to the individual phonation onset flow of the respective elongation and adduction setting, with three step-wise 5 slm increses (e.g., F3 is the onset flow plus 15 slm).

elongation (g)	adduction (g)	flow rate (a.u.)	$v_{mean,sup}$ (m/s)	$v_{max,sup}$ (m/s)	$y_{mean,sup}$ (mm)	$y_{max,sup}$ (mm)	$z_{mean,sup}$ (mm)	$z_{max,sup}$ (mm)	$v_{mean,med}$ (m/s)	$v_{max,med}$ (m/s)	$y_{mean,med}$ (mm)	$y_{max,med}$ (mm)	$z_{mean,med}$ (mm)	$z_{max,med}$ (mm)
10	10	F0	0.12	0.23	0.70	2.35	0.75	1.05	0.18	0.35	0.51	0.85	0.65	1.28
10	10	F1	0.24	0.58	1.11	3.72	1.01	1.48	0.29	0.55	0.77	1.27	0.88	1.58
10	10	F2	0.38	1.08	1.40	3.63	1.36	1.82	0.45	0.88	1.07	1.67	1.22	2.18
10	10	F3	0.36	1.06	1.40	3.46	1.33	2.02	0.52	0.89	1.17	1.94	1.44	2.36
10	20	F0	0.19	0.47	1.08	3.62	1.25	1.39	0.27	0.52	0.65	1.07	0.90	1.72
10	20	F1	0.24	0.61	0.99	2.51	1.32	1.42	0.35	1.11	0.82	1.44	1.00	1.80
10	20	F2	0.35	0.89	1.20	2.72	0.95	1.37	0.39	0.80	0.96	1.55	1.04	2.10
10	20	F3	0.40	1.18	1.23	2.73	0.98	1.42	0.49	1.55	1.03	1.72	1.20	2.20
10	50	F0	0.17	0.42	0.60	1.12	1.01	1.24	0.25	0.49	0.52	0.89	0.71	1.45
10	50	F1	0.30	0.71	1.02	2.33	1.53	1.83	0.45	0.84	0.89	1.49	1.04	2.00
10	50	F2	0.41	1.16	1.43	3.61	1.85	2.00	0.59	1.07	1.14	1.94	1.27	2.25
10	50	F3	0.57	2.04	1.70	3.60	1.75	1.96	0.79	1.50	1.35	2.34	1.42	2.50
20	10	F0	0.23	0.61	1.26	3.35	1.29	1.51	0.30	0.58	0.75	1.32	1.17	2.00
20	10	F1	0.32	0.90	1.32	2.84	1.66	1.76	0.42	1.19	0.99	1.72	1.29	2.24
20	10	F2	0.32	0.93	1.26	2.77	1.17	1.40	0.41	1.64	0.95	1.61	1.24	2.12
20	10	F3	0.38	1.07	1.43	3.11	1.36	1.52	0.50	1.59	1.08	1.79	1.37	2.37
20	20	F0	0.16	0.33	0.72	1.26	0.98	1.23	0.22	0.43	0.53	0.93	0.80	1.46
20	20	F1	0.30	0.97	1.38	3.81	1.62	1.90	0.43	0.83	0.94	1.80	1.28	2.20
20	20	F2	0.35	1.22	0.86	2.21	1.48	1.55	0.52	1.82	0.95	1.64	1.14	2.10
20	20	F3	0.40	1.29	1.03	2.51	0.57	1.42	0.44	1.67	0.95	1.63	1.14	2.10
20	50	F0	0.13	0.29	0.55	1.31	0.78	1.01	0.19	0.37	0.40	0.93	0.64	1.22
20	50	F1	0.24	0.57	0.99	2.02	1.43	1.69	0.40	0.62	0.83	1.54	1.02	1.91
20	50	F2	0.38	1.08	1.32	2.53	1.82	1.96	0.57	1.09	1.11	1.99	1.30	2.21
20	50	F3	0.40	1.25	1.33	2.76	1.58	1.61	0.61	1.40	1.16	1.96	1.25	2.20
